# Context Representation and Fusion: Advancements and Opportunities

**DOI:** 10.3390/s140609628

**Published:** 2014-05-30

**Authors:** Asad Masood Khattak, Noman Akbar, Mohammad Aazam, Taqdir Ali, Adil Mehmood Khan, Seokhee Jeon, Myunggwon Hwang, Sungyoung Lee

**Affiliations:** 1 Department of Computer Engineering, Kyung Hee University, Yongin-si 446-701, Korea; E-Mails: asad.masood@oslab.khu.ac.kr (A.M.K.); noman.akbar@khu.ac.kr (N.A.); aazam@ieee.org (M.A.); taqdir.ali@oslab.khu.ac.kr (T.A.); jeon@khu.ac.kr (S.J.); sylee@oslab.khu.ac.kr (S.L.); 2 Division of Information and Computer Engineering, Ajou University, Suwon-si 443-749, Korea; 3 Department of Computer Intelligence Research, Korea Institute of Science and Technology Information (KISTI), Daejeon-si 305-806, Korea; E-Mail: mgh@kisti.re.kr

**Keywords:** sensors, context, context representation, context fusion, u-healthcare

## Abstract

The acceptance and usability of context-aware systems have given them the edge of wide use in various domains and has also attracted the attention of researchers in the area of context-aware computing. Making user context information available to such systems is the center of attention. However, there is very little emphasis given to the process of context representation and context fusion which are integral parts of context-aware systems. Context representation and fusion facilitate in recognizing the dependency/relationship of one data source on another to extract a better understanding of user context. The problem is more critical when data is emerging from heterogeneous sources of diverse nature like sensors, user profiles, and social interactions and also at different timestamps. Both the processes of context representation and fusion are followed in one way or another; however, they are not discussed explicitly for the realization of context-aware systems. In other words most of the context-aware systems underestimate the importance context representation and fusion. This research has explicitly focused on the importance of both the processes of context representation and fusion and has streamlined their existence in the overall architecture of context-aware systems’ design and development. Various applications of context representation and fusion in context-aware systems are also highlighted in this research. A detailed review on both the processes is provided in this research with their applications. Future research directions (challenges) are also highlighted which needs proper attention for the purpose of achieving the goal of realizing context-aware systems.

## 1. Introduction

In 1991 Mark Weiser introduced initially the concept of pervasive computing [1] that has laid the foundation for context-aware systems [2]. Since then, context-awareness has been in the spotlight. The formal use of context-awareness and investigative research on the term started in 1994 [3]. Pervasive computing strives to provide transparent use of computing facilities to users anytime and anywhere, independently of the environment, whereas context-aware systems focus on providing the right service to the right user at the right time [1–5]. Context-aware systems acquire context, reason on the context and change the system behavior for the user’s changing situation. Context-aware systems adapt their operations and services to the user’s context without explicit intervention from the user. This extra feature of making use of the user’s environment/context for the purpose of computation has increased the usability and effectiveness of such systems and has encouraged many researchers to contribute in this research domain [2,6,7].

Making the context information available to computer systems is the center of attention in context-aware systems [8]. How to develop systems that are context-aware is a key issue to the pervasive computing community [2,6]. The research and development efforts for context-aware systems involve the process of context sensing, acquisition, representation, distribution, manipulation, development support and its implications on human user [2,3,5]. The use of context-aware systems is increasing in variety of different domains, such as location based system [9], context-aware file system [10], context-aware security [11], context-aware activity recognition [6], context based searching [12,13], and intelligent healthcare systems [7,14–16]. Due to their acceptability, the context-aware systems are currently used in many aspects of our everyday life. Various transparent sensing and computing devices are integrated into the user’s environment that acquire user context and process it to provide services to the users based on their context [7,14,17]. Regardless of the context dimensions, *i.e.*, internal or external [18], the general working of such systems is to sense context, acquire it and then make reasoning based on the acquired context [2,17]. This process has pragmatic advantages due to the mature nature of sensing technology used for capturing the environmental context the user is involved in [2,7,14,17].

The internal development approach of context-aware systems is successfully adopted by many systems for their overall implementation. At the same time different researchers have also customized the development approach for their systems’ implementation [6,7,19,20]. This customization mainly includes two aspects after the context acquisition phase, *i.e.*, (1) Context Representation and (2) Context Fusion. Context Representation deals with the formal representation of acquired context for further processing [5]. Context Fusion focuses on the integration of context from different sources in order to merge overlapping and related context information [21]. However, much less attention is given to the process of context representation and context fusion, which are integral parts of context-aware systems or even for the customization of context-aware systems. Context representation and fusion facilitate detecting and recognizing the dependency or relationship of one data source on another to infer user context. This problem is more critical when context information is emerging from heterogeneous sources of diverse nature like sensors, user profiles, and social media at different timestamps based on the user interaction. Both processes are not formally incorporated in the overall architecture of context-aware systems and are not been discussed explicitly for their contribution for the realization of context-aware systems.

In this research we focus on context representation and fusion as the integral parts of overall context-aware systems’ architecture. The needs for these two aspects are highlighted with the help of their use in the existing systems and the amount of attention paid to them [5,7,14,21]. Every context-aware system needs to formally represent the context after acquisition and based on the needs should also fuse it with other related or relevant context. For instance, a context-aware healthcare system needs to formally represent the context captured using sensors to monitor patients’ daily behavior. In case of multiple sensors [7,14,17], the context from one sensor needs to be fused with other sensor’s context to achieve a higher level context with more confidence on the monitored situation [6,8,22]. To highlight the importance of representation and fusion, a detail survey on both context representation and context fusion is presented that discuss the details of these aspects in the context of different systems [2,6,7,17,20].

**Figure 1.**
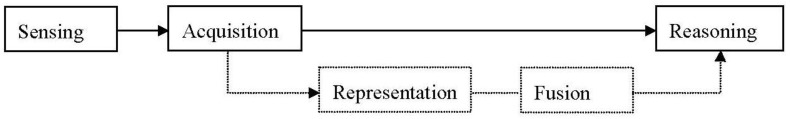
The context-aware systems’ design and development process and our proposed extension to streamline (represented by dotted lines and boxes) the overall scheme by introducing context representation and fusion.

Based on our experience with context-aware systems and based on the existing systems, we propose that representation and fusion aspects should both be addressed explicitly (in sequence as shown in Figure 1) in every context-aware system development. We support our recommendations with a detail survey on relevant context-aware systems, and a detail approach incorporating the two aspects is presented in this research. In addition, we have also highlighted the application of both representation and fusion in the overall process of context-aware system design and development. Still various issues and open research challenges exist in both context representation and context fusion that needs to be address. We have highlighted these challenges (issues that need to be solved) in this research paper for the purpose of achieving the implementation of context-aware systems in its true essence.

Rest of the research paper is arranged as follows: Section 2 discusses context-aware systems and their different components’ functionality in detail. These components include context sensing, acquisition, representation, fusion, and reasoning, which collectively make the context-awareness possible. Section 3 presents a detailed survey on the available context representation schemes used by different researchers in the development of context-aware systems. Section 4 presents a detailed survey of context fusion schemes used and developed by different researchers. Section 5 highlights the applications of context-aware systems based on context representation and fusion aspects. Details on open challenges are also highlighted in Section 5 for both context representation and fusion which needs to be solved for actual realization of context-aware system. Finally, we conclude our research in Section 6.

## 2. Context-Aware System Architecture

**Figure 2.**
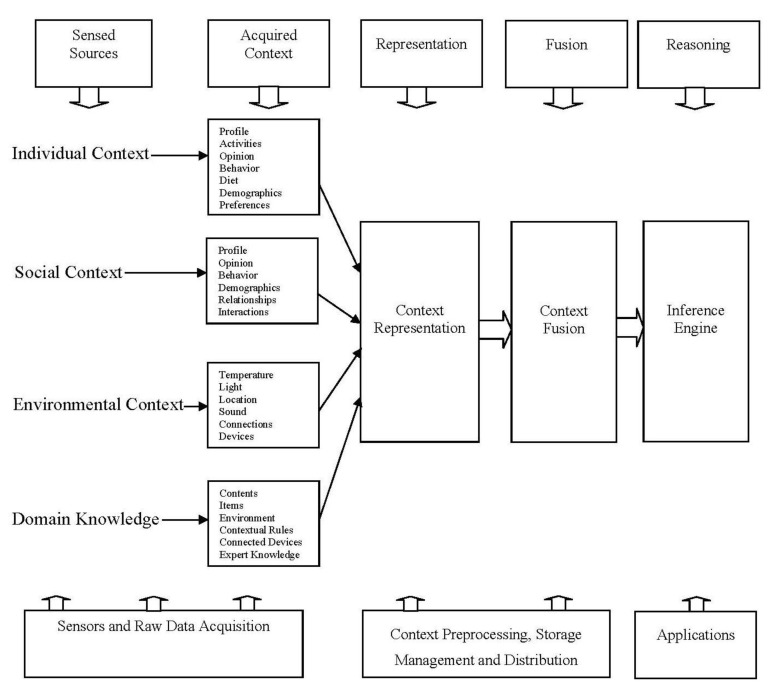
Overall system architecture and components (with associated functionality) of context-aware system design and development process.

In this section we describe the overall conceptual design of context-aware systems, *i.e.*, from context sensing to context reasoning as shown in Figure 2. Various components of context-aware systems and the layers of functionality associated with each component are discussed in this section. The overall architecture of a context-aware system is streamlined and decoupled in this section into the subcomponents of *context sensing, acquisition, representation, fusion, and reasoning* whereas, in existing systems’ architecture they are mixed in three subcomponents, *i.e., context sensing, acquisition*, and *reasoning*. The proposed extensions of context representation and context fusion are also included in the architecture as shown in Figure 2 and their inclusion is supported with relevant research literature. Figure 2 show that how context is extracted from various sources of diverse nature and then processed to give them a unified representation scheme. The representation scheme in most cases is also the storage scheme of collected context [2,6,7]. The next step in Figure 2 is the fusion process where various techniques are followed by researchers to fuse/integrate relevant and closely related context, which can help in making the semantics of context more explicit. On top of the fused context, reasoning schemes are implemented to achieve and develop applications and services for particular needs. The details on research conducted in each sub-component of the architecture (given in Figure 2) are listed in the sub-sections. The main difference in this system architecture is that we have used the notion of acquiring context information from various sources, which are used separately by different researchers for their systems’ customized needs [2,6,18]; however, not used together in a single system. These sources are of vital importance and need to be mentioned and discussed for the overall architecture of a context-aware system design and development.

### 2.1. Sensors and Raw Data Acquisition

A context-aware system relies on the acquisition of information/context about the situation in which the system is operating and about the user who is interacting with the system [2,17,18]. To make the system completely context-aware, the system should be able to capture all the context information from the environment, which a user can perceive. This will decrease the context perception gap between the user and the system, and the system will have the ability to respond according to user intentions [2,7,14].

In many systems, context is solely based on location [2,23] or to some extent the activity a user performs at a given point in time [6]. For such context capturing there are different means of sensing, which are well established. In general such sensing sources are used for monitoring a well structured environment [24], for instance patient monitoring [7,14]. These systems are based on the general user phenomenon of acting and reacting in a given context, which gives a user the capability of adapting with the situation. However, the physical and cyber world offers a richer environment for context acquisition and handling [17,25] that not only help in replicating user action and reaction for an event but also consider user intention, sentiments and preferences for user behavior in a given situation.

#### 2.1.1. Sensed Sources

The sensor technology has major advancements in the recent past that have resulted in significant improvements with respect to physical size, weight, usability, power consumption, functionality, processing requirements, connectivity options, reliability and robustness of sensors [6,17,24]. These studies, developments and the current trend of context-aware systems suggests that it is useful to deploy multiple sensors of diverse nature in environment to sense and capture information about the physical world situation [2,7,14,17]. A list of sensor technologies used to capture user’s physical world context is given in Table 1. Some of these sensors are now readily available in the current versions of smartphones [17].

In many systems, there is a pre-selected criterion for evaluation of the system, so that the trade-offs of the sensing technology used are known beforehand. Only required and useful sensors are used and deployed in the environment for the working system [6,7,17]. Creating a system that is open to new sensors at runtime will further complicate the process; however, it is the real essence of context-aware system to adapt with the changing environment [2]. In addition to a user’s physical world, where users perform different activities and the environment in which the user is; the cyber world where users interact in social media [25] and share their sentiments about different entities [26] is also an important context to be considered for understanding users’ intention, which eventually will help the system to understand the overall context of the user [2,15]. Moreover, in any domain there are rules and regulations that are followed, which are usually encoded as domain knowledge. This knowledge also plays vital role in the successful completion and execution of a context-aware system [7,14].

#### 2.1.2. Acquired Context

Sensor technology is used for user and environmental context, whereas the cyber context is acquired from user cyber activities on social media as well as general usage of computers and smartphones [2,6,17]. The domain knowledge is codded in the system by the experts mostly in the form of rules [14,15]. As seen in Figure 2, most of the context acquired at this step is overlapping with the context acquired using another context acquisition modality. This helps the system to have better accuracy for the context acquired and the confidence on the acquired context is higher for analysis and decision making [2,6]. The context acquired till this phase is in raw representation having no dependency on each other [6,17]. Table 2 shows the type of information captured and used by various systems as context. This acquired context is then mostly used by existing systems at a predefined level for their customized usage. Different machine learning techniques are applied to make the acquired context usable for systems’ customized needs [6,9,27].

### 2.2. Context Preprocessing, Storage Management and Distribution

This section will focus on the representation and fusion of context, which is given less priority in the existing context-aware systems [2,6,7,27]. We believe that proper representation of context is not only important for better preprocessing and storage of context; however, will also help in making the reasoning and analysis job easier. Once a formal representation of context is created then it is easy to distribution and exchange with other storage services and systems respectively [35]. As we know that the acquired context is overlapping and depending on the pre-existing context or post-occurring context, so there is a vital need for context fusion that can facilitate appropriate handling of context for the situation at hand [21].

#### 2.2.1. Context Representation

**Figure 3.**
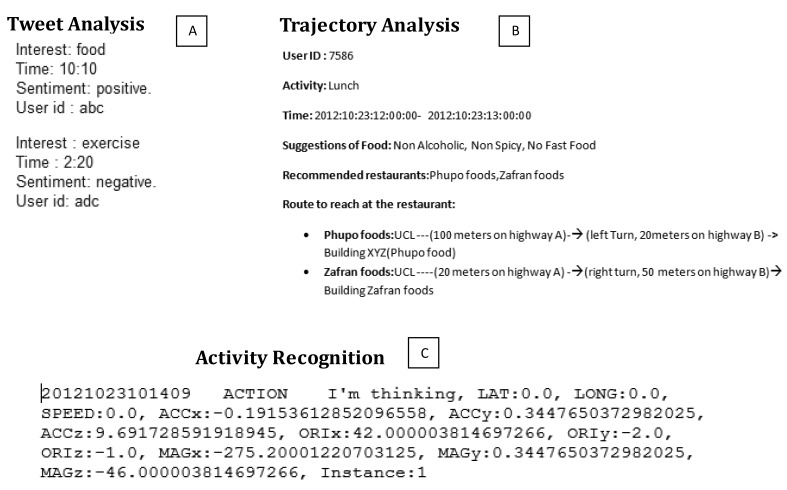
Representation of context information from three diverse input modalities including twitter, trajectory, and smartphone.

After acquiring the context information, the next step in context-aware system is to represent the low level context information captured from different heterogeneous mediums [2,6,7,17]. The limitation of existing context-aware systems is the representation of context information in formal representational format and adaptation of rules that process the represented knowledge [5,7,14]. Until the context is uniformly represented, it is less useful in analysis and reasoning. As shown in Figure 3A–C, there are different possible representations of captured context using Twitter, trajectory analysis and a smartphone, respectively. From Figure 3 it is obvious that all the captured contexts have different representations and they need separate reasoning algorithms to process them. However, if they are converted into a uniform representation format, then it is possible to combine the information in more meaningful manner [2,6,7]. Carneiro *et al.* [36,37] have presented their work on measuring levels of acute stress on humans in online dispute resolution. They analyzed the behavioral patterns of people, when interacting with technological devices. They used a non-parametric statistical hypothesis test to determine differences of features for each user, when they are under stress. This helps in laying down the foundation of a context layer for a virtual environment for conflict resolution. It overcomes the shortcomings of communicating online, which involves the lack of contextual information, such as gestures and body language. Automating user behavior pattern is among the main goals of pervasive services. In [38] the authors present a model for this. They have presented a task model and context ontology, through which, a context-adaptive coordination of services is designed. This helps coordination analysis during design and run-time. The presented software architecture coordinates execution of service according to the current context. Similarly, business processes are also influenced by the context of their environment. In [39] the authors present an approach, named COMPRO (Contextualisation Method for Business Processes), for business process contextualization. It starts from an initial business process model and analyzes it to discover its relevant variations. It then specifies their effect on a business process. COMPRO helps process designers to specify context variants and business process variants that accommodate them. The authors in [40] also emphasize discovering the behavior of users to create intelligent environments, which, in the end, help in understanding and predicting bad behaviors. The same way [41] works on intelligent environment and presents various challenges. The key challenges are: security, privacy, hardware limitations, software limitation to act intelligently, unreliable sensors and networks. According to [42] representation of context and reasoning with context are inherently tied with each other. Therefore the context representation model should consider the efficiency of reasoning during implementation of context-aware systems. In literature, there exist nine different context representation modals that are used in different context-aware systems which we will discuss in Section 3 in detail.

#### 2.2.2. Context Fusion

Context fusion may be required in a system in order to simplify the task at hand, to increase the confidence on acquired context, to identify the redundant data and to reduce the amount of storage space required to save the context coming from various sources [2,8,33,35]. Fusion is required to integrate context information acquired from various sources as the information from one sensor or source may be faulty or error prone [8,32]. This facilitates the goal of context-aware system to reduce the information and computation load on the user of the system. To avoid distracting the user by presenting huge amount of information, the system merges context information from various sources and only presents useful fused information to achieve the end objective [32,43]. For instance, activities like *walking, sitting, standing, running, and jumping* can be fused into a higher level activity of *exercise*.

The context from various sensors may be heterogeneous, so fusion of information is required to understand and merge the information if it is same [6,7,20]. As a large number of sensors are deployed in real world for gathering information from the physical world of users and information is also captured from the cyber world, so there is a need to fuse the context information in order to avoid processing context that belongs to the same category. Otherwise the same context has to be processed multiple times if fusion is not performed [22].

### 2.3. Context Reasoning and Applications

This section will focus on the process of reasoning over fused context and the applications built on top of it. The representation and fusion make the reasoning and application development smoother [2,6,7].

#### 2.3.1. Context Reasoning

Wearable devices have been gaining popularity due to low-cost wireless and sensor electronics [6,17]. There is an increase in the use of such devices for various healthcare applications and for general health monitoring purposes [14,15]. Most of these devices require continuous monitoring of the subject. Furthermore, for many medical conditions, which are hidden, it is important to monitor continuously in order to ensure that no important symptoms are missed [7,14]. The identification of symptoms at times is easy if they follow a sequence, while mostly they are identified using pattern identification and reasoning [2,7].

The system discussed in [7] is based on an ontology for reminder systems and incorporates rules for manipulating the recognized activities of elderly patients in an environment where sensors are deployed to capture the patient’s context. Similarly, in [6], the authors focused on real-time activities recognized using diverse sensors deployed in the environment and for the purpose of reasoning on the context; the authors used these activities, domain knowledge and expert rules for patient situation analysis.

Das *et al.* [44] presented context-aware prompting in smart environments. It has been estimated that by 2040, 23% of US citizens will be of age 65 or older. People with cognitive deficiencies have difficulty in correctly performing the activities of daily life. To help such people, assistive living technologies based on automated reasoning and adoption strategies are gaining popularity. Automated prompting systems like [44] can play an important role in this regard, by using smartphones to deliver prompts or alerts, on-the-go. It has more benefits, as compared to stationary computers or touch screens [17,44]. The context-aware system in [44] uses temporal and environmental information for the determination of situations and then prompts are generated accordingly.

#### 2.3.2. Context-Aware Applications

In [25] the system has processed the social interaction of patients. Based on their social media interactions and activities, the system generates intelligent recommendations. The system presented in [45] was deployed in an Intensive Care Unit (ICU). It was connected to the microbiology lab and patient management system. It is a real-time context-aware system that monitors patients’ daily behavior and symptoms on a daily basis. The system presented in [46] adds context-awareness to the messages passed in the information management system of hospital amongst users (*i.e.*, doctors, caregivers, nurses). It helps the caregivers of a particular patient for whom the context-awareness is added to the message. This also uses the location context by default as this context information is more useful when the caregiver is near the patient or in a patient’s room.

Energy efficiency is also amongst the key parameters of context-aware healthcare monitoring systems. Not all the sensors are required to monitor certain activities like walking, running, and resting. Managing the sensors and other resources intelligently becomes very important [2,6]. Context-awareness has become a viable solution that can help in addressing such issues and alleviate some of the sensing requirements in continuous monitoring. The existing systems are mostly based on one input modality (one type of sensor) and in some cases use imperfect context information [47] for services recommendation. A location-based reminder system introduced in [31] considers location for generating recommendations. HyCare [48] takes context in consideration and develops a schedule for various reminder services. The systems discussed in [7,14,19] use ontology to incorporate context for intelligent processing and understanding user intentions. Using the activities represented in ontological representation is used later for healthcare recommendations and services generation.

In addition to healthcare, context-aware systems are also deployed and used in many other domains. In [49], the authors presented a web contexts classification based on the factors of information quality, which users consider in their minds when choosing websites/web pages. Quality of information consists of various factors like timeliness, information accuracy, reliability, relevance, completeness, and precision. This means that users normally have different information quality factors to consider, while viewing contents. For example, users may be interested not only in the relevance of the content between the hosting web page contents and their information needs, but also in the sources of the contents or the authors of the contents as well [13,49]. Razzaque *et al.* [50] focus on classification and quality of context. They focused on context adoption in heterogeneous environments to the meet requirements of context-aware services. To enable context-aware adaptation, context information must be made presentable.

The systems in [26,51] focused on extracting context about user sentiments from the twitter data. The systems focused on target dependent sentiment analysis for entities of interest to a user. Generally, the user sentiments are classified by the system as: positive, negative, and neutral. Since tweets are short and sometimes not enough for consideration. So, the related tweets are also considered, other than the current tweet. Based on the sentiments extracted using these systems, the system generated appropriate recommendations to user for different products.

Using events as context to trigger the start of context-aware systems is a common approach [52,53]. These systems have direct connection with the captured context and they do not consider the representation and fusion aspects. The starting and stopping of such systems are based on the conditions, if conditions matches then the system will react to the situation in a pre-specified manner. Example of such systems is: warning systems, elevator control systems and smoke detection systems. Context can also enable devices to repeat a behavior for which the system is built [54,55]. Moreover, locating resources particularly depending on the location of resources is also an interesting application of context-awareness [56]. This system is used to automatically detect the printer that is close to the current user.

## 3. Context Representation

In this section of the research paper, we are focusing on the aspect of context representation which is very much neglected in the general concept of context-aware systems’ design and development [2]. Context representation considers different aspects of knowledge processing and reasoning in different phases of context-aware systems. Context information is based on functions, defined in the context modelling approach in a format that can be easily stored, accessed, and exchanged [14]. For example the current context of an object is identified through the reasoning on different context information. According to [42], representation of context and reasoning with context are inherently tied with each other. Therefore, context representation model should consider the efficiency of reasoning during implementation of context-aware systems. In literature, there are different surveys available on context representation models. Hong *et al.* [57] have reviewed five layers of context-aware system, concept and research layer, network layer, middleware layer, application layer, and user interface layer in very comprehensive way. There are different techniques and approaches in each of the above five layers but the authors have missed the internal techniques and approaches. The authors have searched on title, keyword or abstract to find the articles; therefore some articles might have been overlooked in this survey [57]. The authors have covered articles from year 2000 to 2007. After the year 2007 some other techniques of context representations have been published.

**Figure 4.**
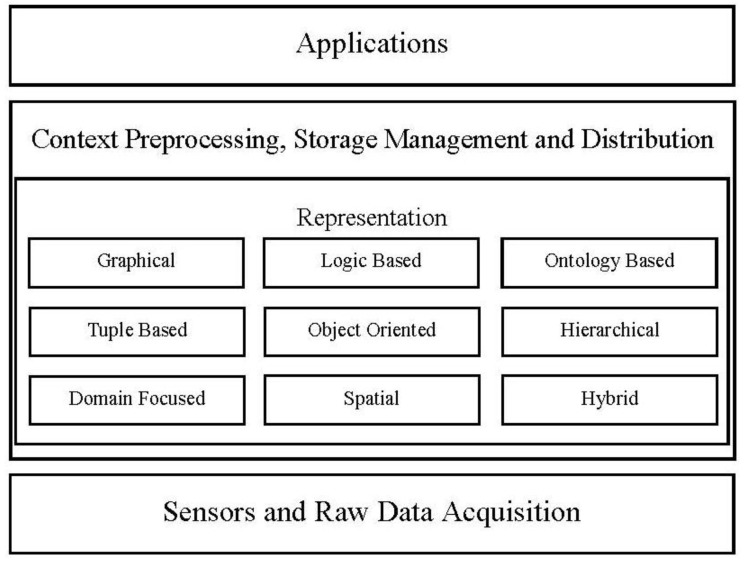
Types of context representation schemes available in literature.

Baldauf *et al.* [2] provided a survey on context-aware systems in 2007 and focused on different context representation models up to year 2005. The authors have investigated five different context representation models that are used in different context-aware systems. Four other approaches of context modeling have been overlooked in this survey. The authors have highlighted that the ontology based representation is a very sophisticated approach but have not discussed model to derive new contextual information and patterns to aggregate new context-aware services. Similarly Miraoui *et al.* [58] have reviewed some context-aware systems up to year 2005; they focused on whole architectures of the systems. According to context modeling, they have highlighted only three context representation models as hierarchical, key-value and ontological models from different context-aware systems. The survey literature provided by Strang *et al.* in [59] has covered surveys on context-aware systems proposed by different researchers before year 2003. The authors have discussed six different context modeling approaches in comprehensive way but the context modeling list is incomplete. Mostly literature surveys on context representation modeling have passed over the Spatial Representation, Hybrid Representation, and Domain Focused Representation models to discuss. A new survey is needed that covers maximum number of models in the latest context-aware systems proposed and developed in recent era. Therefore, we have covered nine different context representation models from latest work on context-aware systems. As shown in Figure 4, there are nine different context representation models/schemes used in different context-aware systems, which we have discussed in the subsections.

### 3.1. Graphical Representation

This context representation scheme includes diagrammatical representation of context at the design time. During development, the graphical context representation schemes need to be translated into a usable format [60]. Henricksen *et al.* [61] utilized graphical context model representation by overcoming the problems with previous models that includes lack of formality and generality [2,53]. The approach tackled issues, such as wide variations in information quality, the existence of complex relationships amongst context information, and temporal aspects of context. The main focus was on features, such as diversity, quality, and complex relationships among context information [61]. The context sources for the scheme considered were: users, hardware and software sensors, and derivation of context from other sources, such as user profile information and user location. Existing context modelling approaches were combined in scenario for the proposed approach [61]. These modelling approaches were: context modelling using existing data modelling techniques and context modelling using object oriented techniques (discussed later). The graph constructs, *i.e.*, entities and attributes were modelled as nodes and associations were modelled as arcs between nodes. There are two challenges in this representation model regarding privacy and distribution of context information. A privacy model is needed to prevent different context information like personal information, location information, and health information by little dissemination. A distribution model is also very important to support the appropriate partitioning and replication of context information in context-aware systems.

The same approach was later extended in [62] by developing Context Modeling Language (CML) as a tool to assist designers in representing context representation requirements for context-aware systems. Main features of the tool were: a graphical representation for describing types of context information (facts, classifications of facts, relevant metadata and dependencies amongst various types of context information), permission of ambiguous context, retaining historical context, enforcement of context, querying database, and expressing situations using a novel form of predicate logic [63]. Their limitations were the flat information model, hierarchical structure that used a particular dominant dimension of context appropriate representations and no support for context exchange with other context-aware systems. From a software engineering prospective, there is a need for the whole software life cycle to associate with context-aware software for better understanding [62]. In this extended work, the limitations of privacy and distribution of context information still need to be resolved.

### 3.2. Logic Based Representation

This type of modelling representation is based on adding context as facts and extracting contextual information using expressions or rules. Formalism is used in these approaches and is often tightly coupled to context reasoning approaches. Gray *et al.* [64] discussed the formal representation of sensed context information in First-Order predicate logic [65]. It also represented the meta-propositional properties, therefore created a hierarchy of context and meta information. Spatial location, time and identity made the sensed context type for representation and properties of the sensed context represented the meta information. This context representation model needs to describe appropriate ways of interactive system functionality relevant to the use of sensed context. This model does not support design and documentation of the design process, due to this limitation this model of sensed context information cannot be used by software tools.

### 3.3. Ontological Representation

Semantic information representation of the contextual information provides the formal specifications and is most appropriate method of context modelling [7,14]. Ontologies provide the base for design of the contextual information representation. Ontology-based models of context information exploit the representation and reasoning power of logics [63,65] for multiple purposes. Interoperability and heterogeneity are two aspects that provide edge to ontological based models over other context representation approaches [6,66]. Petersen *et al.* [67] divided knowledge into two types: domain and generic knowledge. Domain knowledge taxonomic structure consisted of environment context, personal context, social context, task context, and spatial-temporal context. This enabled the system to infer relationships among concepts by constructing context-dependent paths among them. Context space was responsible for storing and retrieving context and was implemented to cover transient and persistent context. It consisted of a context history, current context, and future possible context populated by context instances. This ontological model is using Case-Based reasoning but it needs a feedback system from the user for verification and falsification of results generated by case-based reasoning. The verification process will increase the user confidence on the suggestion provided by the system. The verification of case-based reasoning is still under research area.

Gu *et al.* [68] provided another ontological model for representing context. The model was composed of semantic representation, context reasoning, and context knowledge sharing. The benefit of the ontology-based approach was that context knowledge can be shared among different entities and reasoning about the situation based on the shared context was possible. The procedure included dividing pervasive computing domains into several sub-domains, such as home domain, office domain, and vehicle domain. After division, low level ontologies were defined for each sub-domain. These low level context ontologies were linked up into an upper level generalized ontology. Different context providers acquired various context data from internal physical sensors or external virtual sensors and represented them as context events in the form of Web Ontology Language (OWL) [69]. This sub domain ontological model has limitation of service adaptation during the change of the context. The system is not able to adapt the new service after changing the context of an object.

Horrocks *et al.* [70] proposed an ontological-based approach using OWL that used features from several families of representation languages, primarily including Description Logics [71] and Frames [72]. OWL was primarily designed to represent information about classes of objects and how objects from different classes were interrelated. The emphasis was on the use of OWL as it was particularly important in mobile and pervasive environments, in which different heterogeneous and distributed entities must interact for exchanging users’ context. Importing ontologies that defined by others, into a system is a normal and mostly happening task. Usually a system needs only some specific module of the importing ontology but there is no way in OWL to import that specific module instead of whole ontology.

Chen *et al.* [73] exploited Semantic Web [74] technologies for supporting pervasive context-aware systems. A shared model was maintained in the architecture that behaved as a broker agent for all computing entities. The system then provided a centralized model of context that can be shared by all devices, services, and agents in the space. It also acquired contextual information from sources that were unreachable by the resource-limited devices. It detected and resolved inconsistent knowledge that was stored in the shared model of context with the focus to protect user privacy by enforcing policies that the users had defined to control sharing and use of their contextual information. In [66] the authors proposed standard ontology for ubiquitous and pervasive systems for their information representation in OWL that also included modular component vocabularies to represent intelligent agents with associated beliefs, desires and intentions, time, space, events, user profiles, actions, and policies for security and privacy. The proposed system is facing challenges of abductive reasoning that enables the system to make logical reasoning on different hypothesis. This logical reasoning enhances the ability of reliable observation and explains the relevant evidences. The second challenge to the proposed system is exploring temporal and spatial inferences. Similarly, [6,7] used ontology as a representation scheme for the captured context and manipulation of the context for intelligent monitoring of patients and recommendation generation.

### 3.4. Tuple Based Representation

This type of context model representation is also referred as Key-Value pair [75] and is based on flexible units of data representation. It contains a number of attributes and value mappings as a single record which can be queried using template query [76]. Khungar *et al.*’s [77] system gave special emphasis to group activity and data access rights. It was based on context-based storage that consisted of a logical context data model and physical data storage space. The context information was represented using four concepts: entities, attributes, relationships, and groups. The key-Value representation model is facing a limitation of system performance and user’s satisfaction in mobile computing environment. This representation model needs to enhance the performance of the system in form of proactive retrieval.

Yamabe, *et al.* [78] treated context information as tuple and in current implementation of Citron, the context was represented as *Context: =* {*ID*, *Subject*, *State*, *Time*, *Lag*, *Interval*}. Meta information of the context was containing lag and interval field. Citron has a limitation of performance based on the blackboard architecture. The personal devices become heavily burdened when it analyses the parallel context with multiple sensors.

Bettini *et al.* [79] defined key values pairs for the list of attributes and their values describing context information. Composite Capabilities/Preference Profile (CC/PP) was the first context modelling approach to use Resource Description Framework (RDF) [80] and was considered as representative of key value models and mark-up models. It had limitations in capturing a variety of context types, *i.e.*, relationships, dependencies, timeliness, and quality of context information, whereas allowed consistency checking and support reasoning on context.

### 3.5. Object Oriented Representation

This type of context representation is based on defining context using object oriented programing principles. Abstraction, inheritance, polymorphism, composition, and aggregation are some of the object oriented principles [81] followed by this technique. It followed object’s modelling entities (e.g., people, places, objects) that further implemented context items as attributes or subobjects. Bardram *et al.* [82] proposed Java Context-Awareness Framework (JCAF) which was a service- oriented architecture based context representation system. Context representation in JCAF was performed by making object-oriented models in Java. The core interfaces included were: Entity, Context, Relation, and Context Item interfaces that were implemented by concrete entities/classes. Examples of context used were hospital context and office context. The Unified Modeling Language (UML) diagrams were used for modelling the context. Person, place, thing, and patient were the examples of modelled entities of the system. In addition, *using* and *located* were used as relations among entities.

Mikalsen *et al.* [83] considered context as *any information that can be used to characterize the situation of an entity*. An entity (a person, place, or object) was considered relevant to the interaction between a user and the system. Four types of metadata were suggested in accordance to the system needs: the timestamp (when created), source (who created it), probability (trustworthiness) and user rating (how the user rated the importance of the element). Object oriented representation model needs some further work to guarantee and satisfy the users about the semantics of context used by the services. The proposed system has still a limitation of interoperability and authors suggested the developers to use some 3rd party context management system to overcome this limitation.

### 3.6. Hierarchical Representation

Hierarchical context modelling technique is another approach for representing context using a tree- like structure of context types. Dey *et al.* [84] used this approach to represent hierarchical representation of context to enhance the efficiency of searching in context container. Four essential categories or characteristics of context information were introduced, *i.e.*, identity, location, status (or activity), and time. Identity referred to the ability to assign a unique identifier to an entity, while location was more than just position information in a two-dimensional space. Status (or activity) identified intrinsic characteristics of the entity that can be sensed, while time was context information as it helped to characterize a situation [84]. The hierarchical representation has limitations of privacy and ambiguity in sensed context and its relationships among the different contexts. This representation model needs more work to maintain dynamic environment of the context in the real world. The system should be able to manage the general and traditional context that cannot be sensed directly from the environment.

### 3.7. Domain Focused Representation

This context modelling is based on domain specific information representation. Castelli, *et al.* [85] proposed a general data model for expressing facts by dealing with information coming from various heterogeneous sources. This approach provided ease of querying, processing, and adaptation to context and incomplete information. Facts were expressed by means of 4-fields (Who, What, Where, When) tuple. These were atomic units of factual knowledge also known as W4. “Who” was the user (entered by the user explicitly to the system), “What” was the activity being performed, “Where” was the activity performed (location), and “When” was the activity performed (time). In this “What” and “Where” were derived by the GPS (Global Positioning System), while “When” was provided by PDA (Personal Digital Assistant) or combination of PDA and GPS. The main limitation of this model is less expressiveness and less flexibility. This model needs more flexible strategies for context distribution and access to improve robustness and adaptability in the system.

### 3.8. Spatial Representation

Space is an important context in many context-aware applications and most context definitions mention space as a critical factor [2,7,14]. It is well suited for context-aware systems, such as location based systems [31]. Mobile systems can benefit from this scheme of representation; however, main drawback of spatial context representation is the effort it takes to gather the location data and to keep it up to date [86]. Frank [86] explained the role of spatial context representation in geographical information systems. Consistency constraints were included in all descriptions of the geographic information systems to handle the occurrence of exceptional situations in consistency constraints. Five tier ontology was proposed to depict the relationships among the main constructs of the system. The different tiers proposed were: *Tier 0* human independent realities, *Tier 1* observation of physical world, *Tier 2* objects with properties, *Tier 3* social reality, and *Tier 4* subjective knowledge.

### 3.9. Hybrid Representation

This is a context representation approach that tries to integrate different existing context representation approaches and different types of reasoning in order to obtain more flexible and general systems. Ontological models have clear advantages regarding support for interoperability and heterogeneity that can be easily integrated with other representation schemes, such as Spatial, Tuple Based, and Logic Based representations. Henricksen *et al.* [87] proposed a hybrid approach by using reasoning and interoperation. This type of approach provided a hybrid solution that combined interoperability support and various types of ontology-based reasoning. The hybrid approach was based on a mapping from customized representation constructs to OWL-DL classes and relationships [87]. The problem of this representation is inappropriate choices that arise from reliance on flawed context information that reflect the user’s requirements. The second problem is privacy, preferences information is exposed to the users.

### 3.10. Critical Review

Context representation and reasoning on context information are integrally tied with each other [42]. The context-aware systems use different types and diverse nature of information like event information, environment information, user information, and temporal information to provide different kinds of context-aware services. Therefore, context representation should consider different aspects of information representation and it should select a modeling approach in a format that can be easily used to store, access, and exchange the information. There are different context representations models have proposed in literature and have been used in different context-aware systems. The graphical representation model proposed in [61] overcomes the lack of formality and generality in the previous context representation models. In previous models there were other issues of handling the wide variations in information, complex relationships in context information, and temporal information of context. The graphical representation in [61] overcomes these types of limitations. The graphical representation provides a formal basis for representation and provides easy way for reasoning on the diverse context information. This proposed model also handles the communication channels for the context users; therefore privacy and distribution of context information are very important for such type of representation model. A privacy model is needed to the system for preventing context information like personal information, location information, and health information by dissemination. Therefore, a distribution model along with privacy model plays important role to support replication and dissemination of context information among the different context-aware systems.

The *Logic Based Representation* model presented in [64], for expressing requirements for sensed context information in terms of relevant quality attributes of context as well as the properties of the sensors that provides the context information. The sensed context information can easily be formulated using *Logic Based Representation*. The context information composed into complex expressions and associated with meta-propositional properties. This model can identify and manage the quality information of coverage, resolution, accuracy, repeatability, frequency, and timeliness. This model has a limitation to describe appropriate ways of system functionality relevant to use of sensed context. This model cannot use by any software tool due to deficiency of supporting documentation of design process.

The *Tuple Based Representation* model presented in [77] is a very easy model to represent the context information in key-value pairs. The system represents the context as entities, attributes, relationships, and groups in easy manner. But this representation model needs to enhance the system’s performance and user’s satisfaction in the form of proactive retrieval. *Ontological Representation* provides base for designing contextual information representation and handles the semantics among different context information [14]. *Ontological Representation* exploits the representation and reasoning power on complex and diverse nature of context information. Similarly this representation model enhances the interoperability and heterogeneity aspects of representing context information. The *Ontological Representation* Model presented in [67], provides the adaptability of user interaction, anywhere at any time. This model also offers automatic situation assessment through case-based reasoning. Case-based reasoning gives a closest solution rather than an exact one, therefore a feedback system needs to ensure the exact solution and enhance the user’s satisfaction. According to [7], the ADL (Activities of Daily Living) ontologies provide a model that establishes the semantics between activities and contextual information using activity-based properties. Context ontologies easily streamline the whole process of collecting and managing low-level sensor data, transforming to middle-level information fusion, and to high-level activity recognition. This ontological model needs to recognize the concurrent activities of multiusers and further ability for handling the temporal information such as sequence and duration. Horrocks *et al.* [70] have proposed ontological representation model to represent diverse context information that interrelate with semantics in mobile environment. This model appropriate to represent heterogeneous and distributed entities, interact with each other for exchanging the user’s context. The proposed system imports the required whole ontologies defined by other context-aware systems rather than a partial ontology, when it needs.

The *Object Oriented Representation* model based on defining context using object oriented approach and it follows all the object oriented programming principles. This approach handles the super class and sub class relationships among different context information. The Java Context-Awareness Framework proposed in [82] is service oriented architecture based context representation model to represent information about context, relation, and context items in object oriented manner. This model needs to enhance the interoperability by increasing the semantics among different context information. The *Hierarchical Representation* of context information enhances the efficiency of searching the context [84]. Likewise the Object Oriented model, it handles the context information in the form of super class and sub class only in hierarchical manner. This model cannot handle all the semantics and relationships and it needs to enhance the maintenance of dynamicity in environment of the user’s context.

The *Domain Focused Representation* model deals with context information coming from difference sources. It provides the easiness in querying the context, easily process, and adapt the context and incomplete information. The context information represents in an easy way of 4-field tuples. Due to 4-field tuple representation, the model has low expressive power and less flexibility. The *Hybrid Representation* model integrates the important properties of different representation models to obtain more flexible and general model. The ontological model has properties of expressiveness and heterogeneity that can easily integrate with other representation models. The problem of this model is inappropriate choices that arise from reliance on flawed context information that reflect the user’s requirements. The second problem is privacy, where preferences information is exposed to the users.

### 3.11. Discussion

According to the review of context representation schemes presented above, it is observed that context representation models should fulfill the maximum requirements of knowledge representation. Validation of context data to the schema is one of the requirements of context representation model. Similarly, the unique identification of context some times and in some scenarios plays important role in the reusability of context. A reliable and useful context representation model appropriately handles uncertain and incomplete information provided by different sensors. Likewise the generality, expressiveness and expandability are other requirements of a context representation model [5]. None of the above mentioned schemes tackled all these requirements; however, they tried to cover the requirements that relate to the system and context environment of their domain and needs.

Some context models represent simple and small context data in very efficient ways [79,84]; however, they were not able to represent complex data. The ontological representation [6,66,67] is very expressive approach to represent complex data and provide complete semantics and relationships among different context information. Due to more expressiveness, the context information is easily sharable and reusable among different sources. The ontological models also handle different types of heterogeneous data that comes from various sources. The ontological models check the consistency of relationships among the context information. According to our study the ontological representation is becoming a de facto standard in interoperability among different systems. On the other hand, there is a little support for handling the temporal values in ontologies. There is also lack of fuzziness and limited handling of uncertainty in existing ontological representations.

The hybrid approaches usually perform very well in most of the cases. In [47], the hybrid approach combined the benefits and important characteristics of ontological representation with other representation schemes. It provided a reasonable combination of expressive power and efficient reasoning power. From the overall survey, we have observed that imperfection of context information is handled in very few systems and up to a very limited scale. Some systems have handled it with partial satisfaction conditions only.

## 4. Context Fusion

In context-aware systems the behavior or response of the system depends upon the historical context maintained and the recent context captured by the system [2,7,14,27]. Various types of data coming from different sources can be treated as context and the behavior of the system is modified in accordance with the context. The focus of this section is on the context fusion for context-aware systems where context fusion is a process of integrating context about an entity into a consistent and useful representation [2,7,8,30,33]. Different systems use different techniques and algorithms for context fusion. The most commonly used approaches are: weighted sum, product, min, max, probabilistic, fuzzy, and ontological. Table 3 presents a summary of techniques used to achieve context fusion. They can be broadly classified into two categories: Probabilistic and Logic based. Probabilistic approach includes genetic algorithms, neural networks, Bayesian networks, and Dempster-Shafer theory. Logic based approaches use basic mathematics, set theory, and past knowledge to logically infer relationships among entities. Ontological approaches use semantic technologies to fuse information coming from various sources. These approaches are discussed separately in upcoming sub-sections.

### 4.1. Context Fusion Based on Probabilistic Methods

This section presents an overview of various context fusion techniques that are based on probabilistic methods. Among all probabilistic methods Bayesian analysis was found to be the technique preferred in most context fusion researches [28,29,33,34]. Machine learning techniques such as neural networks [22], and genetic algorithms [20] were other probabilistic techniques used in development of context fusion systems. A hybrid approach combining probabilistic methods with other context fusion methods is also utilized to provide context fusion [22,28,33]. For example a weighted sum or product approach is used with probabilistic method by assigning proper weight to the probability term and using this term in weighted sum or product formula. The upcoming Subsections 4.1.1 to 4.1.6 provide detailed discussion on context fusion systems that use probabilistic methods.

#### 4.1.1. Dynamic Weighted Information Fusion

The World Wide Web is an important source of information. The amount of information is so huge that it is becoming an increasingly difficult task to locate relevant information for a specific user need. Results obtained by using any search engine, e.g., Google, also depends upon on whether the user is experienced or novice. In USearch [28] context-aware information fusion was used to provide more accurate results to a user of web search engine. The *query enhancement* [28] module rearranged user’s query based on the context information available. Then this modified query was passed to Google and the results returned from it were reordered based on context of searched information.

The context information was provided by the user explicitly (e.g., interests, rate contents) or implicitly (e.g., Google Calendar). In order to add context information to the user query, location information was added using Google Calendar or Microsoft Outlook. Personal vocabulary added by the user was used to substitute terms in user query. Different types of searches were possible based on the selection from the user. Options other than web search were map search, people search and restaurant search.

Three approaches were adapted by USearch for information fusion, *i.e.*, weighted sum of products, Bayesian analysis and combined approach. In weighted sum of product approach a score of 0 (irrelevant) and 1 (relevant) was assigned based upon the context. With the passage of time USearch also did further personalization by observing and analyzing the pages a user visited. Peer information and recommendations were also considered for personalization of search. In Bayesian analysis only title and summary of the page was analyzed to find the probability score. It was assumed that if more relevant terms were found in the page then the page was considered most probably relevant. In combined score method basic sum of products formula was used; however, the Bayesian score was also added to it with proper weight assigned to it. In USearch a combined method was preferred as it provided more improvement in performance.

#### 4.1.2. Context Fusion for Vehicle Safety

As reported in [21], 57% of the time human error was the only reason for accidents and 95% of the time human factors contributed in accidents. The field of information fusion for vehicular and driver safety has been explored in recent research [21,88,89]. Current active vehicle safety systems do not take context information into account, e.g., is the driver distracted or not? So the authors in [21] addressed this issue and came up with context-aware driver system. The system consisted of three sub-systems: Driver ID sub-system, Maneuver ID sub-system, Distraction detection sub-system. Three maneuvers were considered in this system: left turn, right turn and lane change. CAN-Bus (Controller Area Network-Bus) vehicle dynamic signals were used. They include the steering wheel angle, steering wheel speed to identify the distraction level of the driver and also to recognize the maneuver. Gaussian Mixture Model (GMM) and Universal Background Model (UBM) were used in all the three sub systems mentioned above. The system [21] successfully performed maneuver recognition and distraction detection along with driver identification by using GMM and UBM.

The systems’ [21] working was described as follow. In the first step the driver of the vehicle was identified. After this identification the system only focused on a single person. A maneuver recognition system identified the maneuvera performed by the driver which were then linked with the identified driver. The distraction detection system performed an assessment of distraction level of the user based on the signals received from the sensors. The result of the distraction detection is then fused with pre-captured context for prompts and recommendations to the drivers.

#### 4.1.3. Context-Aware Fusion of Gait and Face for Human Identification

In [22] context-aware fusion of human gait and face was utilized for identification of a person. Most of the biometric sensors that were available those days worked on static fusion rules. Static rules for biometric information fusion were not able to incorporate the context information and resulted in loss of accuracy and performance of those systems [6,7,14]. Face recognition performance depended upon the condition of lighting in the area where the system was deployed. In [22] two factors were considered (*i.e.*, distance between the subject and the camera and view angle) that can affect the performance of the system.

As an application, fusion of gait and face for a human based on context-awareness was presented in [22]. Human gait and face were obtained from a single source and for testing two datasets were used. For facial recognition a front view was preferred while in case of gait recognition the side view was considered more important as it captured more motion characteristics [90]. In order to fuse information for a person in case of static fusion, a function mapped the biometric feature vector to the fused information for that individual. The static fusion was based on score level or feature level. In score level fusion it determined the matching score for a specific vector with the database that was already available to the system. The function that mapped the biometric traits to the fused information was fixed and was unaware of the context. This issue is eliminated in [22] using context-aware information fusion function that not only was depending upon the biometric traits, but was also depending upon two additional inputs: the perception signal which represented the context at a specific time and the prior knowledge about how the fusion rule was affected by the external condition. The perception captured how the context was changing with respect to time and the prior knowledge highlighted how the fusion rules were adjusted to incorporate this change. The fusion of information occurred at three different levels, match score level, feature level and decision level. Usually matching at score level was easy. Static rules were one of four types: sum, product, min or max. The context-aware fusion of human gait and face was based on the levels of scores.

For fusion of context information there were two possible approaches for implementation: weighted sum approach and machine learning approach, and both were investigated in [22]. Machine leaning approach was based on neural networks. It was reported that machine learning approach was superior as compared to that based on prior knowledge (weights).

#### 4.1.4. Context-Aware Filter Fusion for Face Recognition

In [20] context information was used to enhance the accuracy of facial recognition systems. Currently the performances of the facial recognition system depend upon many factors like bad illumination and pose angle, distance between the camera and the subject [90]. Bad illumination is the major cause for the degradation in performance. Context-aware filter fusion was used to get better image quality in the presence of bad illumination condition [20]. For images captured under bad lighting conditions the recognition rate dropped as the edges of the images were blur.

Structure and parameters of the filter were reorganized and rearranged based on the context in which the image was captured. Fuzzy ART (Fuzzy Adaptive Resonance Theory) was used to cluster the images according to the illumination. After clustering of images in accordance with the condition of illumination the images were sent to the filter block. Three types of filters were applied: Retinex, Histogram analysis, and Contrast Stretching. For adaptive filter fusion genetic algorithm approach was used. The fitness function used in [20] for genetic algorithm was based on the weighted sum approach. Weights were assigned for system correctness and class generalization.

#### 4.1.5. Information Fusion for Intelligent Environment

Context-aware information fusion for intelligent environment was presented in [29]. The system focus was energy conservation in People Help Energy Savings and Sustainability (PHESS) [91]. The intelligent environment was able to gather information about the user, and store it, and then future decision and/or analysis were performed on this information. The information fusion’s concept was that information was gathered from different sources and it was used to improve the accuracy and quality of information that was coming to the system in future. Instead of using physical sensors, virtual sensors were utilized in accordance with the sensor fusion strategy. The system architecture consisted of the following layers: sensor layer, model layer, and reasoning layer. Sensor layer reduced some undesirable characteristics, such as false senor reading and garbage values due to sensor’s inaccuracies. On the basis of gathered context and context fusion, a model was created that captured the behavior of attributes. This model was used to estimate the current state of intelligent environment and predicted the next state.

#### 4.1.6. Sensor Fusion for Context Understanding

The purpose of sensor fusion is to understand the context in human computer interaction which was discussed in [33]. The sensing process was broken down into two steps: first, context information was broken down in the form of facts and quantitative measurements so that a model could be built, Second, generalized sensor architecture was purposed to gather the information coming from highly distributed sensors and then fuse the information. This information was then used to populate the information fusion model. To gather the context from the user or environment, sensors were deployed; however, there were many problems due to a number of reasons, such as different resolution for different sensors, errors in reading and redundant data from different sensors.

The proposed architecture in [33] was tested in a conference room environment. Functions, location of conference room, facilities, and usage policy were predefined and available to the system. Sensor fusion was used to map the raw data gathered from the sensors to the relational database that represented context. Georgia Institute of Technology Toolkit [92] was used with the sensor fusion mediator module designed by authors to manage the uncertainty from the sensors. Each sensor sent data as captured data and confidence. Afterwards, based on the confidence level of various information sources the information was fused. The extension to the work was presented in [34] that also discussed the relationship between the Dempster-Shafer theory and Bayesian method for the purpose of information fusion. The author approach was compared with weighted sum of probability method (discussed above). They represented context information by discrete symbols and numbers. The mappings from raw sensor data to the context representation were well defined. The goal was to manage the information overlap and resolve conflicts/inconsistencies. The system demonstrated that this theory was closer to human observation, perception and the reasoning process.

### 4.2. Logic Based Context Fusion

Logic based methods provides context fusion by inferencing relationships between data in a context-aware system. They either use mathematical relationships to identify relationships and merge data [32] or use knowledge from past experience [8,30]. Both types are separately discussed in the next two subsections.

#### 4.2.1. Context-Aware Information Fusion for Intelligence Analysis

Jenkins *et al.* [8] described how information coming from soft data sources (human agents) and hard data sources (sensors) can be combined to produce better results in counter insurgency intelligence operations. In intelligence analysis, there were many data sources and the analyst faced problem in understanding and analyzing all information. It was hard to infer relationships among different information and infer meaningful results from it. The main goal of all this process was to come up with the complete picture and understand how different factors were connected and affected each other. Fusion of information from different sources reduced the load of information and helped in analysis. Hard data sources had predefined and known error characteristics, whereas in soft data sources the error characteristics were hard to find due to unpredictability of human behavior. In many situations the incoming information from soft data sources were depending upon the context in which the soft data sources were located. Information fusion took a number of factors in to account while fusing the context information coming from various sources. In the system, estimation of age was presented as an example (for detail please refer to [8]) of information fusion. The context information used for fusion to estimate age were: target and observer race, observer training in age estimation, availability of target facial cues, observer’s age in relation to target age, and known possible range of target age [8].

#### 4.2.2. Information Fusion in Healthcare

A context-aware information fusion for healthcare systems was discussed in [30]. A multi-agent system was proposed that located a user in a given context. With the availability of cheap commodity sensors various characteristics of a user and the environment around user were captured by deploying many sensors in observation area. The system was intelligent to switch off un-utilized resources in environment. The context was collected using sensors and interpreter agent performed the data fusion among other tasks like reasoning to extract higher level context from the gathered context. The systems discussed in [7,14] also used sensors to collect the micro level context about patients in an environment. The micro context was later interpreted with ontology and expert knowledge in rules to infer higher level context and generate recommendations to the patients.

#### 4.2.3. Information Fusion for Avoiding Ship Collision

Collision avoidance between ships was discussed in [32] by incorporating the context information available. The system consisted of two types of agents: a ship agent and VTS (Vessel Traffic Services) agent. A multi-agent model was utilized for solving the problem as they normally work in distributed and heterogeneous environment. The system [32] focused on information fusion for collision avoidance and fusion was achieved at three fusion levels: original information fusion, multiple union fusions and distributed plan fusion. Location information in the form of longitude and latitude, speed and course information of current ship were used for fusion. At multiple fusion level, for making collision avoidance plan other ships in the vicinity were also considered which were considered as risk for the current ship. At distributed plan fusion level inconsistences between planes made by individual ships were also considered and then resolved.

### 4.3. Critical Review

Suitability of context-aware fusion technique in an application area depends upon the requirements and constraint specific to that area. Every fusion technique has certain advantages when used for an application, whereas it may pose some problems when it is used in some another application area.

Probabilistic methods provides an increase in fusion performance and accuracy [20–22,28], but they also suffer from some limitations. For example in case of Bayesian analysis, prior probability must be known in advance. In order to find the prior probability one must have a large dataset to come up with a realistic estimate. A less accurate estimate can severely degrade the accuracy of Bayesian analysis. Similarly in machine learning techniques, it is necessary to train the system before it can be used [21]. This requirement necessitates the use of two separate datasets: training dataset and validation dataset. So, large amount of data is required in order to train the system before the actual use. Nevertheless, once an accurate estimate/model is obtained, probabilistic methods generally perform better as compared to static fusion methods [28].

Logic based models rely on logical inferencing on fusion model [8,30,32]. Relationship between data is described with the help of mathematical model. In simple scenarios where relationships among data sources can be established with ease and all the factors linking different data sources are known in advance, this approach is very useful [32]. In many realistic scenarios, the underlying relationship between data sources is too complex to represent mathematically [8]. For instance, in applications that involve human judgment, it is very difficult to model human response as it varies greatly from one person to another and depends upon his knowledge and past experiences. In order to establish relationship among data sources in such cases error characteristics can be used [8], which are based on a large dataset from the past and also involves psychological human behavior. Problem with this approach is that it is very specific to one application only. If an error characteristic function is formulated for one application, e.g., age determination, in most cases it is practically impossible to extend it to other application. A separate error characteristic is needed which gives an accurate estimate for that application. It is recommended using this approach when information about the relationships among data sources is known in advance. Also this approach is helpful when there is human involvement and a model for human response is available. This approach may become difficult to use in case of complex scenarios having complex relationships among data sources and when model representing human behavior is too complex to draw some inference within the available time constraints required for the application.

Semantic web technologies (ontological models [7,14]) are also used to provide context-aware information fusion. Ontologies are made up of many tuples that show the relationship between two elements connected by an arrow representing the relationship between them. The ontology can be used to infer the connection among different entities in the model through reasoning based on some logic. It is also possible to merge two or more ontologies together to get a more detailed model representation. There are huge domain specific ontologies available to use. For example in the domain of healthcare SNOMED CT (Systematized Nomenclature of Medicine Clinical Terms) [93] and ICD-10 (International Statistical Classification of Diseases and Related Health Problems) [94] are available. Like every information technique, ontological technique also has some disadvantages. First of all, it requires a lot of time to create ontology from scratch. Large amount of data has to be processed. After creation of ontology it is also required to validate the ontology and check it for consistency. Ontological models for information fusion rely on the finding the relationship between entities. This requires a large amount of data to be processed before finding the answer to the query and establish the link between different entities. This issue makes it less suitable for real-time applications. There are also few limitations for natural language processing if the query is in natural language.

### 4.4. Discussion

In order to provide context-aware information fusion in web searching, context information is used to modify the query and also rearrange the results [28]. The purpose of using context-aware information fusion is to display the most relevant results to the user in accordance with the current context. The main challenge in this approach is to integrate context information with the user query. One way is that the user explicitly provides his context, which is not a very preferable approach. It requires the user to have a basic understanding of the overall system which may not be the case most of the time. Contrary to this method, user context can also be inferred by using the available information e.g., search history. In this case there is a risk that the user context might not be incorrectly inferred by the system and as a result the results returned may be more irrelevant as compared to the case where fusion is not used. Conflicts can be resolved using certain methods, but in this case there is a critical issue of time required to find the relevant information instead of the value of information. Going through the whole process: query enhancement, web search, rearrangement, and conflict resolution may require time that exceeds the value of the requested information.

Context-aware fusion also finds its application in the domain of security. Biometrics is by far the best method to provide human identification in security applications. Biometrics include: human face, gait, fingerprint and retina. In context-aware human face recognition systems context information is used to provide a better recognition rate for human face and reduces the effect of factors that degrade the performance of the system. For face recognition, factors that affect the performance of systems are: illumination [20], distance between the camera and the human, pose angle [22], *etc.* The human face recognition information can also be fused with other modalities like human gait in order to have better recognition accuracy for a person [22]. The most important part in any face recognition system is the process used to capture an image or a video. In the case where the image/video accusation is not performed in a controlled environment the results of face recognition systems are less accurate. Context-aware information fusion provides an efficient solution to this problem. For instance, if the illumination condition is bad, it can be detected and filtered to get a better face recognition result [20]. Combining face recognition information with other modalities is motivated by the fact that the accuracy of most face recognition systems is low when they are used in uncontrolled real environment. Human face information can be fused with the gait information to provide a system usable in uncontrolled real environments. Fusion in such cases makes use of either static or dynamic fusion rules [20]. Static fusion rules are less accurate but simple to implement. Dynamic fusion rules are more accurate; however, they require training as in most cases they use machine learning approach. In order to train the system a large dataset is required. This approach suffers from the same limitations as other probabilistic approaches discussed in Section 4.3.

In addition to other application areas mentioned above, healthcare is another major application area for context-aware information fusion. Context-aware information fusion is used in assistive technologies for elderly, helping doctors in diagnosis and treatment of diseases, and designing smart homes. With the availability of cheap commodity sensors it is becoming easier and easier to monitor vital signs from a human body. In addition, evolution of smartphones and integration with sensors provides a powerful platform to monitor a patient’s health with ease. For activity recognition of patients sensors are deployed in the environment. Fusion of data is done in order to infer high level activity. Each activity consists of several low level activities, e.g., cooking consists of using stove, cutting, chopping, washing *etc.* There is no fixed order of such low level activities and the user can follow them in any order. This makes it difficult to fuse data and logically infer the high level activity. One way is to use the low level activity data and construct ontology from it. Then it becomes easier to infer high level context and/or activity information from it.

## 5. Applications and Challenges

In this section we discuss the usability/applications and future challenges for both context representation and fusion. The applications highlights the importance of both aspects in the overall design and development process of a context-aware system, whereas the challenges provide the directions to research community for further research and development in context representation and fusion areas to solve the unresolved issues. Applications and challenges for context representation are discussed first and then followed by discussion on context fusion.

### 5.1. Context Representation Applications

This sub-section discusses the applications of context representation. Three applications of context representation, *i.e.*, Context Modeling, Context Analysis and Adoptive Systems, are discussed which are important to consider while designing a context-aware system.

#### 5.1.1. Context Modeling

The context modelling is important for both storage management and exchange [2,6]. A context model was proposed in [61] that was useable in flexible pervasive computing applications. The context representation model and infrastructure facilitated the programming of applications to gather, manage and disseminate context information to applications and achieved the concept of interoperability [35]. This also facilitated in a range of management tasks, such as integration of context information from a variety of sources, management of sensors and derived context, detection of conflicting information [61].

The authors in [73] proposed a shared model of context for all computing entities in a given space and enforced the privacy policies defined by the users when sharing their contextual information. It was based on ontological representation of context information and mainly used for privacy protection mechanism in an intelligent meeting room. Similarly, in [66] an ontological model facilitated intelligent agents with associative beliefs, desires, intentions, time, space, events, user profiles, actions, and policies for security and privacy, whereas, in [77], context model for efficient storage management of the context was proposed. The model was used to manage the user data/context and make it available to that user based on his/her situations and contexts.

An object oriented context model was proposed in [82] for context representation that facilitated in creating context-aware systems. The same model was also used in *context-aware hospital bed*. In this system hospital bed adjusted itself and reacted according to entities in its physical environment like patient, medicine and medical equipment. It was also used in *wearable computers for emergency personnel* system. It helped them react to changes in the work context. In [85] a domain focused model for context representation was proposed that facilitated the users in gathering information about the world they interact with and understanding it. One of its applications was to automatically build and maintain tourist diary. The second application facilitated a group of users to share their actual GPS locations with each other.

#### 5.1.2. Context Analysis

In [64] analysis on sensed context information was formulated to help in design and implementation of context-aware systems. The sensed context information was represented in form of First-Order Predicate Logic and the reasoning was achieved with the help of predicate logic based inference engine. In [86] the authors developed the context representation in ontology and analysed the context at five different levels, such as human independence, assessment of the physical world, entities with interrelationships, entities with their interrelationship in social domain and knowledge on a subject in a specific domain. The hybrid representation [47] facilitated communication among applications that assists the users with the selection of appropriate communication channels for their interactions with other people.

The ontological representation of patient context in [6,7] was used to monitor situation and analyse user history for the possibility of abnormal patient behaviour. The represented and new acquired context was also analysed with expert knowledge encoded in rules to suggest and recommend services to the patients. Working with the system for better representation and prompting users for their activities was also achieved in [17].

#### 5.1.3. Adaptive Systems

Ontological representation of context was also used to produce adoptive systems [67] to the user based on user situation. The system was demonstrated in a multi-agent system for supplying context-sensitive services in a mobile environment when user was moving around. A similar system was also produced in [68] that focused on applications and services that must be more aware and adaptive to highly dynamic environments (intelligent vehicle environment). The context-aware system proposed in [78] used tuple based context representation to capture and store user’s context information and user’s surrounding environment. This context information was later used by the system to adapt the behaviour of application running on the personal device to user context and facilitated user with appropriate services.

A dynamic decision support system was proposed in [16] that represented the context captured from various sources in ontology. Based on the newly captured context, the system analysed the user situation and also at the same time checked for possible changes in the system behaviour for user context. So if changes were detected then the systems’ internally stored context was updated to adapt the system behaviour to user’s context.

### 5.2. Context Representation Challenges

In this sub-section the challenges or the open research issues related to context representation are discussed that needs proper attention and research and development efforts for their solution and eventually for the realization of context-aware systems.

#### 5.2.1. Heterogeneity

Heterogeneity is one of the important factors of a context representation model [2,14]. In context-aware systems the input context comes from diverse sources like various types of sensors, videos, users profiled data, derived data and context from social media. The context representation model should handle such type of heterogeneity and present a unified model to represent and accommodate the diversity of contextual information.

#### 5.2.2. Mobility

Mobility is another requirement to consider in context representation modelling. The mobility exists in context-aware applications due to running on mobile devices [17]. On the other hand some context-aware systems depend on mobile context information sources as well [79].

#### 5.2.3. Expressiveness and Reasoning

A context representation model should facilitate representation, inference and storage of complex context including entities and relationships among those entities [2,14,35]. This capability makes a context-aware system more expressive that allows very flexible reasoning on context information; however, time complexity of reasoning may increase due to more expressiveness [6].

#### 5.2.4. Imperfection

Due to heterogeneity of context it is possible that redundant and noisy context may end up in the system [47]. To transform the noisy context into usable context it is possible to drop some valuable context information or it might cause other dependent context inconsistent [6]. Therefore the context representation model should tackle the inconsistency, incompleteness, uncertainty in heterogeneous context.

#### 5.2.5. Simplicity, Reusability and Extendibility

Simplicity, reusability and extendibility are interrelated with each other. Simple representation of context can facilitate reusability of context and that also facilitate the extendibility of both context and context-aware systems [79]. The system should also support the interoperability of context information from one system to another.

#### 5.2.6. Timeliness

The context representation models should facilitate a persistent context history. This history helps in access the past behaviours as well as the system can infer about the future behaviour and status of the system. In context-aware applications the management of context history is difficult due to continuous updating of context information and changing behaviour of user and system [7,35].

#### 5.2.7. Relationships and Dependencies

Relationships play an important role in handling the dependencies of one context information on another one and cover the complete semantics among context information [6,7]. The semantic depicts and ensures the correct behaviour of the context information captured by the context-aware applications [79]. Therefore, the requirements and features are very important to consider while designing the context representation model.

#### 5.2.8. Representation Standard

The main issue with all the representations discussed above is that there is no standard representation for context [2,7,14,16]. Even the most prominent and better suited context representation scheme (*i.e.*, hybrid scheme [47]) lacks in standardization. This restricts the context-aware systems from porting context information of one smart space to another.

### 5.3. Context Fusion Applications

This sub-section will focus on the applications of context fusion. Four applications of context fusion, *i.e.*, Reducing Information Overload, Context Fusion for Identification, Sensor Data Fusion and Context Fusion in Healthcare are discussed.

#### 5.3.1. Reducing Information Overload

The current web is a huge repository of web documents and searching relevant information from it is a challenging task [13]. The authors in [28] introduced a fusion technique for improvements in search results retrieved by a web search engine for a particular user. It showed that more accurate web search results can be obtained if user’s context information was used either to add additional contextual information to the query entered by the user or to rearrange the web results retrieved by search engines. This helped to reduce the information load on user. Similarly, Silva *et al.* [29] worked on the development of middleware for intelligent environments for the purpose of reducing the information load by using techniques from context-aware systems’ domain. Intelligent environment collected information about user, and stored it so that future decision and/or analysis can be performed on this data. Information fusion’s concept was that information is gathered from different sources and it was used to improve the accuracy and quality of data that was collected.

#### 5.3.2. Context Fusion for Identification

Current biometric fusion systems adapt itself with the change in environment conditions because they are based on static fusion rules. The systems proposed in [20,22] used video context and human gait, fused it together to identify human users based on their unique characteristics. On the other hand, [32] proposed a fusion technique that combined information from various sources at multiple levels to identify the possibility of ship collision and if such situation is about to occur then adopt avoidance strategy.

#### 5.3.3. Sensors Data Fusion

A transportation safety system was proposed in [21,95] that fused the context information coming from sensors, context information about driver and information about the wheels turnings. This information was fused and used to generate safety alarms. In [8] a context-aware approach was used to add observation gathered by human intelligence into fusion process of information from both hard and soft context sources about various factors. Human behaviour is very dynamic and may vary from one person to another. Observation about one human may or may not be generalized as it depends upon a number of factors and context about these factors are fused together that provided better analysis of the factors involved [8].

#### 5.3.4. Context Fusion in Healthcare

Context fusion technique presented in [30] used context-aware agents in healthcare domain. A multi-agent architecture used to collect patient information and then fused it for patient monitoring purpose and intelligent analysis of patient context for a time period. Relatively same approach was also presented in [33]. The sensors’ collected context information was fused to produce better understanding of the user in a room. Later the work was extended in [34] by using Dempster-Shafer Theory for sensor fusion to improve the overall performance of the system.

The context information collected using various sensors deployed in the patient’s environment was also fused in [7,14] to monitor user behaviour and user daily life activities. Both the systems used ontology based fusion process to achieve better understanding of patient context and then provide appropriate recommendations. In [16], the authors proposed to learn from the fused information about the change in patient behaviour and then change the system behaviour to provide up to date and appropriate services.

### 5.4. Context Fusion Challenges

In this sub-section, the challenges or the open research issues related to context fusion are discussed that needs to be solved in order realize the true essence of context-aware systems.

#### 5.4.1. Process Standardization

There are several different approaches followed for the purpose of fusion (*i.e.*, probabilistic approach [96], weighted sum approach [34,97], fuzzy set based approach [95] and ontology based approach [7,14]). These approaches have various advantages and disadvantages. There is not a single uniform or standard fusion approach which is acceptable and applicable in different systems. This also poses challenges in interoperability and portability of context from a smart space to another. In addition, the accuracy of current context fusion approaches is marginal [21,29,32] and that needs improvement.

#### 5.4.2. User Identification

Context is collected from various different sensors, social media, and environment. The problem during fusion process is to identify and relate exact user to the context collected. For instance, a smartphone will collect context about the user using it; however, the sensors deployed in an environment are collecting information about all users in that environment. Detecting which context is for which user is a difficult task and then merging/fusing it with other collected contexts without identifying the corresponding user is not possible. Another problem for cyber context is that a user may have various social media accounts and with different IDs. Fusing context about a user in this case is again an issue of first identification of user and associated accounts.

#### 5.4.3. Time-Sparse Context Fusion

At time there are situations where context produced at two different time intervals are of relevance and must be fused. For instance, in the morning a user share context on social media that he/she will perform exercise at 3:00 pm. Then at 3:00 pm, the sensors collect user exercise context. Both these context information shared in the morning and detected at 3:00 pm needs to be fused; however, there is no system that considers such cases. In another case it might happen that the user might forget to perform exercise, now the morning social media context is no more valid so must not be considered. However, there is no fusion approach to work for such situations. These cases are better applicable in healthcare monitoring systems and have various applications in healthcare domain.

#### 5.4.4. Assessing Confidence Level of Different Modalities

Context-aware information fusion is based on data received from a number of sources. For example, in case of intelligent environment systems, many sensors are deployed to monitor physical world quantities, e.g., light intensity, heart rate, and body temperature. Due to the distributed nature of such context-aware systems, the data on which the current context is based on may be outdated. There are number of reasons for this to happen: (1) There is some time required to gather data from all sources during which the physical environment may change. (2) Some sensors may give false readings. (3) Some sensors may be offline due to low power levels. In such scenarios, there is an additional burden on a context-aware information fusion system to take such factors into account and infer a context that is recent and give a reasonably accurate depiction of the physical environment. One possible way to overcome this problem is to gather some extra information from the source, which gives a measure for the confidence for the current data. The confidence level can be used in fusion process to assign an appropriate weight to the reading.

#### 5.4.5. Storage Management

Collecting context information from a diverse and larger number of sources poses a challenge of data storage. Large memory is required to process and store the data gathered from various sources. A framework can be used for providing efficient storage management [29]. For instance, in healthcare systems, sensors are deployed to monitor vital signs of a patient. In most cases the data is sent to a central location and it is fused to get the information about context. Storage of information for a single patient may not be a huge burden in terms of memory requirements; however, consider a scenario where this system is implemented in a hospital environment to monitor all the patients. It will require fairly large memory storage. Also retrieval of information from such large storage is another issue. Some proposed solutions include: data filtering, data mining, and efficient storage techniques [98]. Storage management is another area that requires attention from the research community.

#### 5.4.6. Maintaining Privacy

Maintaining privacy of a user is most important aspect of any modern systems. Context-aware information system is also required to maintain the privacy of a user. In order to fuse information together, data is gathered from many different sources. For example, a driver wants to know the traffic situation in a specific area. Let’s suppose that in order to present the relevant information to the user the system must fuse location and speed data from all the sources in a specified area to infer traffic status. It will be required by the system to track the location of all the users all the time. It becomes a cause of concern if any user is not willing to share his location data. This problem can be solved by using a trusted third party; however, this approach also has its pros and cons. Other possible solutions include: Adaptive Data Anonymization [99] and information security techniques in order to protect the user information [98].

#### 5.4.7. Degree of Human Involvement

Context-aware systems have an inherent responsibility to reduce the information load on a user. More sophisticated algorithms and programming is needed when there is less human involvement [24]. In ideal scenarios, it is required to present the user only with relevant information and with minimum feedback from the user. Consider an example application to keep track of an internet user’s likes and dislikes for a web surfing scenario. It will be very annoying if the application require a user to rate every webpage he visits by displaying a pop up window every time. One way to solve this problem is to ask the user explicitly to set the level of involvement, which is preferred by the user. In some cases human involvement is very critical. In information fusion for medical data, it is necessary to have a fair level of human involvement. In short, human involvement in any application depends upon the requirements of the application and the cost related to the case where a wrong fusion is performed by the system without the user involvement.

#### 5.4.8. Real-Time Information Fusion

Some applications require data to be processed in a very small amount of time, e.g., intelligence analysis [8]. The processing capability of computing resources has increased many folds over the last few years. Still the processing capability of small sensory hardware, e.g., motes, is low as compared to desktop computers, so generally, there is a processing delay when data is processed in such hardware systems. In addition, there is also delay in gathering information and transmitting it from one place to another. After collecting data, it is processed to get relevant information. Processing large amounts of data requires a longer duration. This results in a delayed output from the system. Information fusion based on ontology also requires doing processing over an entire ontology. All such procedures results in a longer delay which poses a problem in real-time application scenarios. Removing data redundancy and amount of data can help achieve the goal of real-time information fusion in context-aware systems.

#### 5.4.9. Data Redundancy

When data is gathered from a large number of sources, there is a possibility that the data may have certain level of redundancy. For instance, in a smart home environment there are a large number of sensors deployed. In order to provide complete coverage of an area the sensing range of neighboring sensors overlap with each other. It results in data redundancy as the data is gathered from the same location. Some processing can be done to detect and discard the redundant information. It provides benefits in terms of information storage at the cost of more processing. One other possibility to reduce redundancy is to deploy a hierarchal data gathering infrastructure. Data should be checked for redundancy at every level of hierarchy. This approach is beneficial since it requires to process less volume of data at a time. Preprocessing can also be done to reduce the data redundancy [100].

## 6. Conclusions

Context-aware systems are widely used in various domains and context-awareness and are becoming an integral part of every system. The acceptance of context-aware systems and their functionalities are the main reasons for the current research focus in this area. However, there is very less attention given to the process of context representation and context fusion, which act as backbone of a context-aware system to have better interpretability. Both the processes are followed implicitly in some research work conducted; however, these are not been discussed explicitly for the realization of context-awareness and also not discussed collectively for their effective usage in context-aware system. We have explicitly focused on the importance of both context representation and fusion by referring to the existing literature and the scheme available. For their effective usage, we have streamlined their existence in the overall architecture of context-aware system and have highlighted their use. Different applications achieved with the help of context representation and fusion, when formally incorporated in a context-aware system, is also discussed in this research. A review on both context representation and fusion, alongside future research directions and challenges in the field are highlighted. These challenges need proper attention for the purpose of achieving the overall goal of context-aware systems.

## Figures and Tables

**Table 1.** A list of sensor technologies used for context acquisition.

**Sensors**			
Accelerometer	Temperature sensor	Position sensor	Level sensor
Gyroscope	Humidity sensor	WiFi source	Motion detector
Video sensor	Air pressure sensor	Light sensor	Touch sensor
Audio sensor	Bio sensor	Binary sensor	Soft sensor
Location sensor	Weight sensor	Vibration sensor	Heartbeat sensor
Touch sensor	Electronics sensor	Free fall sensor	Colorimeter
Motion sensor	Magnetic field sensor	Digital sensors	GSM source
3D images and video	GPS	Depth sensor	Speed sensor
Body sensor	Temporal sensor	Water sensor	Chemical sensor
Smartphone	Smoke sensor	Gas detector	Image sensor

**Table 2.** Context captured and used by various existing context-aware systems.

**System**	**Contexts used**
Nicole *et al.* [28]	Google calendar, location, Bayesian analysis, areas of interest, personal vocabulary, personal information, information about friends and colleagues
Sathyanarayana *et al.* [21]	Steering wheel speed, steering wheel angle
Anderson *et al.* [22]	Distance between camera and subject, view angle
Khattak *et al.* [6]	Teeth brushing, eating, walking, in living room, taking medicine, reading book, watching TV, exercise
Young *et al.* [20]	Illumination
Jenkins *et al.* [8]	Error characteristics of soft and hard data source
Han *et al.* [17]	In the bus, in the subway, walking, running, cycling
Silva *et al.* [29]	Luminosity, temperature, humidity
Tapia *et al.* [30]	Light, smoke, temperature, doors’ states
Sohn *et al.* [31]	Location
Liu *et al.* [32]	Latitude, longitude, course, speed, risk
Wu *et al.* [33,34]	Voice recognition, focus of attention, pose angle
Chen *et al.* [7]	Walking, making coffee, using sugar, hot water, taking cup
Fatima *et al.* [25]	Social interaction, keywords, blood sugar level
Khan *et al.* [27]	Walking, running, moving up-stairs, moving down-stairs

**Table 3.** Existing systems and the context fusion scheme used by these systems.

**Type**	**Reference**	**Method**
Probabilistic	Nicole *et al.* [28]	Weighted sum of products, Bayesian analysis and combined approach.
	Sathyanarayana *et al.* [21]	Gaussian Mixture Model (GMM)/Universal Background Model (UBM) and likelihood maximization learning scheme.
	Geng *et al.* [22]	Sum, Product, Min, and Max, Machine Learning (Neural Networks)
	Young *et al.* [20]	Genetic Algorithm
	Silva *et al.* [29]	Bayesian network
	Wu *et al.* [33,34]	Dempster-Shafer theory of evidence

Logic/Ontology Based	Chen *et al.* [7]	Ontology and rules based
	Khattak *et al.* [14]	Ontology and description logic rules based
	Liu *et al.* [32]	Mathematical functions, Reasoning
	Jenkins *et al.* [8]	Fuzzy membership function transformation
	Tapia *et al.* [30]	Interpreter agent
